# Interactive Video Simulation for Remote Healthcare Learning

**DOI:** 10.3389/fsurg.2021.713119

**Published:** 2021-08-10

**Authors:** Dahlia Musa, Laura Gonzalez, Heidi Penney, Salam Daher

**Affiliations:** ^1^New Jersey Institute of Technology, Department of Informatics, Newark, NJ, United States; ^2^College of Nursing, University of Central Florida, Orlando, FL, United States

**Keywords:** healthcare simulation, interactivity, video, engagement (involvement), teamwork, authenticity, remote learning, nursing

## Abstract

Simulation is an essential component of healthcare education as it enables educators to replicate clinical scenarios in a controlled learning environment. Simulation has traditionally been conducted in-person through the use of manikins, however, the COVID-19 pandemic has challenged the practice of manikin simulation. Social distance constraints were enforced during the pandemic to reduce the potential spread of the virus and as a result, many educators and students were denied physical access to their universities' simulation facilities. Healthcare educators sought remote alternatives to manikin simulation and many resorted to instructional videos to educate their learners. While the use of videos increases safety, passively watching videos lacks interactivity which is an important component of simulation learning. In response to these challenges, we developed an interactive video simulation software that uses educators' existing video content to conduct a simulation remotely, thereby promoting safety during the pandemic while also meeting the interactivity standards of best practice for healthcare simulation. In this paper, we compare the interactive video simulation to the current practice of watching non-interactive video of a simulation using the same content. We found that interactivity promotes higher order learning, increases teamwork and enhances the perception of authenticity. Additionally, the majority of participants demonstrated positive reception of the interactive simulation. The simulation software provides the safety desired of a remote simulation during the pandemic while also engaging students in interactive learning experiences.

## 1. Introduction

Nurse education technology and strategies are constantly changing, leading researchers in nursing education to identify the best ways to teach learners. Knowledge acquisition and learner engagement are requisite skills of nursing education (1, 2) and simulation has proven to be a very important strategy to achieve these skills (3). Simulation facilitates learning and promotes patient safety; it affords nursing students the opportunity to experience realistic replications of clinical cases and practice their skills without impacting the condition of a live patient (4). The use of simulation increases students' self-efficacy which is indicative of competence in the clinical setting (5). Additionally, repetition is an important component of learning nursing skills (6). Unlike actual clinical cases, simulation experiences can be infinitely repeated under the same or similar conditions.

Simulations are traditionally conducted using a manikin, a lifelike patient simulator that represents the whole or partial human body (7). Other simulation methods include standardized patients (SPs) and virtual simulations. SPs are human actors trained to play the role of a patient in a scenario, and virtual simulation allows students to apply knowledge and practice skills in a virtual recreation of reality (7). Traditional manikin simulation is a well-recognized nursing education strategy, however, it has limitations regarding visual fidelity (8), cost (1), and now safety. Manikin simulation requires students to interact directly with the manikin while an instructor oversees the simulation; therefore, students and instructors need to be present concurrently within the same space. Due to the cost of manikins and space restrictions, typically students enter the simulation in small groups. The restrictions enforced in response to the COVID-19 pandemic have made it difficult for nurse educators to safely conduct manikin simulations due to limited space, high manikin costs, and safety. Many schools of nursing have grappled with identifying quality clinical substitutions that could be experienced remotely. Nurse educators need the flexibility to be creative without space constraints (2).

In many healthcare educational institutions, educators adopted virtual technologies to continue providing simulation to students during the pandemic. Commercialized virtual simulation programs, such as Second Life (9), vSim (10), and Shadow Health (11), have been one virtual option. Second Life enables students and instructors to interact as avatars within a virtual world that depicts clinical environments. In Second Life, educators develop the virtual environment and simulation experience themselves, empowering them to design the most effective simulation experience for their learners. Second Life has been shown to promote positive learning outcomes in nursing education and to particularly impact students' collaboration and engagement (12). In a study by Beyer-Berjot et al. (13), Second Life was used by surgical educators to develop a comprehensive simulation when other virtual simulation options did not encompass all components of the curriculum. Second Life has demonstrated to be a beneficial tool for healthcare educators, however, it is complex. While development may be feasible for some educators, others need to hire developers to create their simulation or rent another user's virtual environment. In vSim and Shadow Health, students assess digital standardized patients in pre-developed simulations. Students have responded positively to the use of these virtual simulations and recommend their use (14), but the rigidity of the systems presents limitations to educators who are unable to modify or expand on the lessons. Telesimulation is another remote learning option that has recently been more widely adopted by nursing educators. Telesimulation refers to the use of telecommunication technologies to provide simulation experiences to learners in a distant location, typically where immediate access to the simulation facility is unavailable (15). In a telesimulation model developed by Naik et al. (16), students learn COVID-19 ventilator management by viewing a tutorial video and then joining a telesimulation session hosted by their instructor via a video conferencing application. Students in the telesimulation watch as the instructor performs ventilation on a manikin according to their instruction. This method of using video content to conduct remote simulation works as a low-cost replacement to in-person simulation and has been used by many educators during the pandemic. In our study, we used this method for control. Virtual platforms, such as Microsoft Teams, have also been used to support remote educational activities as well as facilitate collaboration and cultivate a sense of community during the pandemic (17).

Like many other schools of nursing, we had to pivot to ensure our learners met the expected curricular outcomes while learning in a remote environment. We recorded exemplar videos of a nurse instructor performing scenarios in a simulation laboratory and showed the videos to students remotely. The exemplar videos demonstrated the ideal conduct of the scenarios which students would have performed themselves if they had access to the simulation facility. The use of simulation videos, however, played a role as a mediocre replacement to in-person simulation activities. Interactivity is an essential component of healthcare simulation (18) and passively watching simulation videos without interactivity does not satisfy the standards of best practice for healthcare simulation (19). To resolve this issue, we developed an Interactive Video Simulation (IVS) software that converts a simulation video into an interactive experience using educators' existing video content. After we developed the IVS, we evaluated the validity of our software as a remote alternative to traditional simulation. We asked the following research questions:

**Q1:** Does **interaction** with the simulation system promote **higher order learning**?
**Q2:** Does **teamwork** promote **higher order learning** compared to individual work?
**Q3:** Does **interaction** with the simulation system increase the perception of **authenticity**?
**Q4:** Does the **order of participation** affect **perceptions** of the simulation?

## 2. Materials and Methods

### 2.1. Study Design and Procedure

This section describes the design and procedure of the study as approved by the Institutional Review Board (IRB). In a mixed design study (between participants, and within participants) nursing students were split into teams of two participants at a time and were asked to participate in two different simulations: an interactive (INT) simulation, and a video (VID) simulation. The schedule was pre-allocated without prior knowledge about the students. All participants experienced both modalities with a unique scenario each time, but in a different order. The two scenarios (Stroke and Chest Pain) were included in both simulations. Participants were exposed to each of the simulations once and participated in both scenarios. For example, if a student viewed the Stroke scenario in the VID simulation, they would view the Chest Pain scenario in the INT simulation and vice versa.

#### 2.1.1. INT Simulation Condition

**Setup**

**Figure 1:**
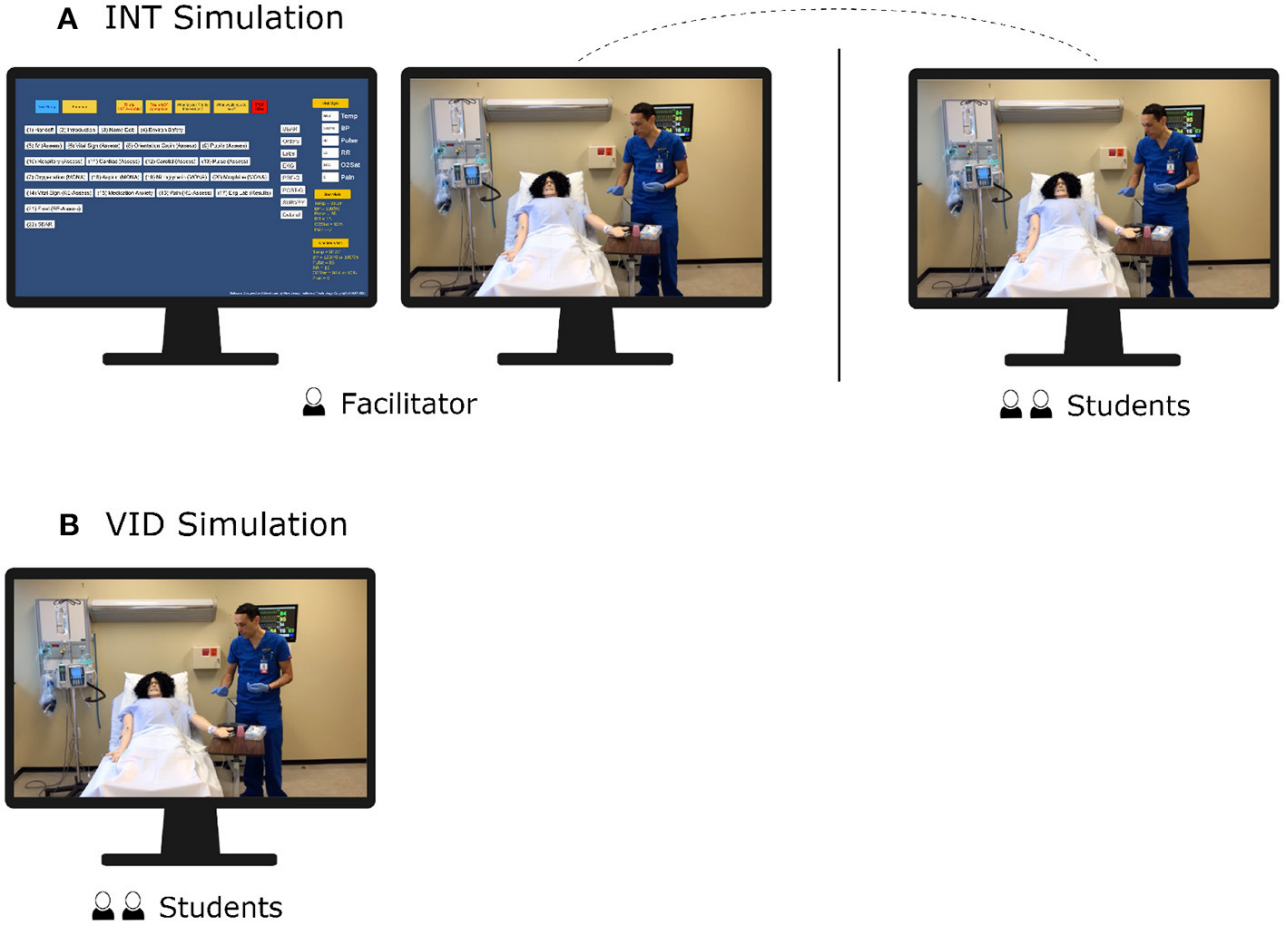
This diagram shows the setup of the interactive simulation and video simulation used in this experiment. **(A)** For the INT simulation, the facilitator shared the content-controlled screen to the students via Zoom. **(B)** For the VID simulation, students watched the non-interactive video content.

The INT simulation was delivered through the use of the IVS simulation software that we developed. The facilitator ran the software on their computer using two screens: one that displayed a dashboard of buttons to control the simulation, and one that displayed the simulation content. From a remote location, the facilitator shared the screen displaying the simulation content with the students over Zoom. The screen displaying the dashboard of buttons was not visible to the students. The facilitator used these buttons to control the simulation content viewed by the students on the other screen. The students connected to Zoom from a computer in their classroom, where they watched the simulation content shared by the facilitator. The setup for the INT simulation is shown in Figure 1A.

**Procedure**

Students reviewed the Scenario, Background, Assessment, and Recommendation (SBAR) for 3 min and then had 4 min to complete the pre-questionnaire. After completing the pre-questionnaire, students participated in the INT simulation for 20 min. The facilitator asked the students to describe their nursing care interventions in the order they would perform them, and as a team, students described the steps of their patient care. For each step described by the students, the facilitator played the corresponding video clip on the shared screen showing a nurse performing the step. If the step described was not available as one of the video clips or was not appropriate, the facilitator displayed a message saying “This option is not available” or “This option is not appropriate,” respectively. The facilitator also displayed lab images (EKG, CT, lab results), vital signs and the SBAR as needed by the students. By the end of the simulation, the students had made the decisions for the patient's care and watched videos of a nurse delivering care to the patient. The IVS software generated automated logs of the teams' answers during their simulation. Since the video clips displayed were the ones described by the students, it is possible that the students did not view all of the video clips. After the simulation, students had 4 min to complete an individual post-questionnaire and 5 min to complete a survey. Participants were then debriefed by the facilitator for 10 min.

#### 2.1.2. VID Simulation Condition

**Setup**

Students watched a non-interactive exemplar video in a classroom. The video depicted a nurse performing interventions to care for a patient in the given scenario. The video was a concatenation of all the video clips that we recorded for the scenario. The facilitator played the video for the students and remained in the classroom with the students for the duration of the simulation, though the facilitator did not need to control any aspect of the simulation. The setup for the VID simulation is shown in Figure 1B.

**Procedure**

Students reviewed the SBAR for 3 min and then were given 4 min to complete the pre-questionnaire. Then, the students watched the exemplar video for either the chest pain or stroke scenario without interruption. Students watched as the nurse in the video provided the ideal sequence of care without their input. After watching the video, students were given 4 min to complete an individual post-questionnaire, and following the post-questionnaire, students discussed with each other and had 8 min to complete a team questionnaire. Students were then given 5 min to complete a survey. Lastly, students were debriefed by the facilitator for 10 min.

### 2.2. Software Design and Development

The IVS software is intended to be used by healthcare instructors to engage students in virtual simulations. We developed the IVS software in Unity (20) using the language C#. The software requires two screens: one screen displays a dashboard of the simulation controls for use by the instructor, and on the other screen the students view the simulation. The simulation content (videos, images, vital signs, and the SBAR) is imported into the software, and for each piece of content, the software creates a button which the instructor uses to control its display. To generate these buttons, the software retrieves information from databases that identify the button specifications. The instructor has the ability to enter the databases and make modifications to customize the software as needed by editing CSV files. To create the video and image buttons, the CSV files take data such as a button label, the associated file name and the order of the button on the screen as input.

The software is designed to separate the controls from the content to hide the options from the students while allowing the content to be shared over a video conferencing application, such as Zoom or WebEx. The frame rate of the videos is 30 fps but could be reduced depending on the connection of the video conferencing application. The instructor can share the content-controlled screen to the students over the video conferencing application while the other screen remains visible and accessible only to the instructor. During the simulation as students describe the steps of their nursing care, the instructor uses the button controls to display the content depicting those steps, simulating the students' delivery of the patient care. The software maintains a log of data identifying which buttons were pressed and what information was displayed throughout the simulation. The instructor can later review the log file to see the students' sequence of steps and the amount of time they required to decide each subsequent step. This technology can be used both in groups and with individual students.

### 2.3. Multimedia Content

For this study, we recorded a series of video clips that depicted a nurse at an American university performing two simulation scenarios. In one scenario, the nurse assesses a stroke patient and in the other scenario, the nurse determines the cause of a patient's chest pain and intervenes. Each video clip that we recorded shows the nurse performing an individual step in the scenario. There were 40 video clips in total, each in the MP4 format, with a frame rate of 30 fps and resolution of 1,920 × 1,080. Eighteen of the videos were for the stroke scenario and 22 were for the chest pain scenario.

The content for the INT condition included the video clips, images, and vital signs and an SBAR as text. For the VID condition, the video clips were concatenated back-to-back to form one exemplar video for each scenario. The clips were ordered so that the nursing steps would be shown in the ideal sequence. The length of the video for the stroke scenario was 15 min, 10 s, and the length of the video for the chest pain scenario was 16 min, 9 s.

### 2.4. Healthcare Scenarios

We chose two different patient care scenarios to conduct this study. The scenarios are part of the curriculum for the university's nursing program. Both patient scenarios required students to not only implement basic patient safety into their care, but also specific care or protocols that are necessary for positive patient outcomes for the different diagnoses. The scenarios involved the care of a stroke patient and a chest pain patient.

#### 2.4.1. Stroke Scenario

The first scenario was nursing care of a patient named Vera Real with a cerebral vascular accident (CVA) or stroke. Along with completing patient safety interventions, the students were challenged to perform a comprehensive neurological assessment, identify hypertensive crisis, and administer appropriate prescribed medication. The scenario also included options to review laboratory data, review radiology data and also update the admitting provider as part of the simulation.

#### 2.4.2. Chest Pain Patient

The second scenario was a patient named Anne Marie with complaints of chest pain and anxiety. This scenario challenged the students to determine if the chest pain was cardiac related or anxiety related and to intervene as appropriate. Safety interventions (hand hygiene, patient identification, and room safety) were also necessary for successful completion of the simulation. The students had options to administer oxygen, cardiac medications, and/or anxiety medication. This scenario had options for the students to review laboratory data, review an electrocardiogram (EKG), and contact the admitting provider.

### 2.5. Participants

The participants of this study consisted of 36 undergraduate nursing students at an American university. Of the 36 students, 32 identified as female, 3 as male, and 1 participant did not identify as either. In terms of ethnicity, 19 students identified as white, 12 as Hispanic or Latino, 3 as Asian, 1 as Black, and 1 as both Asian and Latino. All students had previous simulation experience. Thirty-four students had experience using mannequins and 32 students had experience with virtual simulations. All students were in at least their fourth semester of the nursing program. Two participants had incomplete data: one participant was missing a pre-questionnaire and team questionnaire and the other participant was missing a team questionnaire. These participants were excluded from tests that required the missing data.

### 2.6. Measures

During the study, participants completed pre-, post- and team questionnaires which all included the question: *After reviewing the SBAR, outline your nursing care in the order you would perform them and without omitting the basics (e.g., start with introducing yourself to the patient)*. The pre-questionnaire was asked before the simulation and the post-questionnaire was asked after the simulation. The team questionnaire was asked following the post-questionnaire and was completed only by the VID condition. Team responses for the INT condition were collected from the data logs that were generated automatically by the IVS software during the simulation. The data logs for the INT condition and the team questionnaires for the VID condition were considered equivalent measures of team learning in our data analysis. The pre- and post-questionnaires were submitted as free text, as was the team questionnaire for the VID condition.

At the end of the study, participants completed a survey that measured two aspects: perception of teamwork and perception of the authenticity of the encounter. We used questions from the Self-Assessment Teamwork Tool for Students (SATTS) shown in Table 1 (21). These questions were included in Factor 1: Teamwork Coordination and Communication, and Factor 2: Information Sharing and Support, of the SATTS questionnaire. Participants answered the SATTS questions on a Likert scale from “poor” (1) to “excellent” (7). We also measured the authenticity of the simulations using questions from the Virtual Patient Evaluation (VPE) shown in Table 2 (22). These questions were included in Factor I: Authenticity of Patient Encounter and the Consultation, and Factor II: Cognitive Strategies in the Consultation, of the original VPE questionnaire. As the original questions in Factor I were intended for medical students, we modified the questions to be applicable to nursing students. Participants answered the VPE questions using a Likert scale from “strongly disagree” (1) to “strongly agree” (5).

**Table 1:** Self-assessment teamwork tool for students (scale from 1 to 7).

SATTS1: Role	Each team member had a clear role.
SATTS2: Plan	A plan for treatment was communicated to the team.
SATTS3: Communication	When team members received instructions they closed the communication loop.
SATTS4: Instructions	Instructions and verbal communications were directed.
SATTS5: Overview	An overview of the situation was maintained.
SATTS6: Suggestions	Suggestions were invited from within the team when problem-solving.
SATTS7: Assistance	Team members offered assistance to one other.
SATTS8: Situational info	Situational information was verbalized.
SATTS9: Teamwork	Overall teamwork.

**Table 2:** Virtual patient evaluation questions (scale from 1 to 5).

	While working on this case…
VPE1: Decisions	I felt I had to make the same decisions a nurse would make in real life.
VPE2: Nursing care	I felt as if I were the nurse caring for this patient.
VPE3: Gathering info	I was actively engaged in gathering the information (e.g., history questions, physical exams, lab tests) I needed to characterize the patient's problem.
VPE4: Revising image	I was actively engaged in revising my initial image of the patient's problem as new information became available.
VPE5: Summarizing problem	I was actively engaged in creating a short summary of the patient's problem using medical terms.
VPE6: Nursing priorities	I was actively engaged in thinking about which findings supported or refuted my nursing priorities.

In the last question of the survey, we asked participants which simulation technology they preferred, either video or interactive, and why. Participants also could optionally provide any comments about the technology they used.

## 3. Results

The data was not normally distributed and therefore we needed to use non-parametric statistical tests (23, 24). The Mann-Whitney *U*-test was used for analyzing data between subjects and the Wilcoxon signed-rank test was used for analyzing data within subjects.

### 3.1. Learning

To gauge participants' learning, we analyzed the data of the pre-, post- and team questionnaires. We downloaded and prepared the questionnaire data for analysis, and then sorted participants' responses into categories using components of the Quality and Safety Education for Nurses (QSEN) competencies (25): assessment (A), intervention medication (IM), intervention communication (IC), evaluation (E), and safety (S). We assessed participants' learning at the level of these categories. The data was analyzed by comparing participants' responses to the correct sequence of videos. As many videos included more than one step, participants received credit if they identified the main significant step performed in the video. The post-questionnaire and team questionnaire were differentiated by the fact that participants completed the team questionnaire with their partners and the post-questionnaire individually. To reflect this differentiation, we will refer to the team questionnaire as the team response and the post-questionnaire as the individual response in this section and following sections. The data was analyzed for the scenarios separately as each scenario incorporated a different sequence of steps.

#### 3.1.1. Scenario 1: Chest Pain

**Figure 2:**
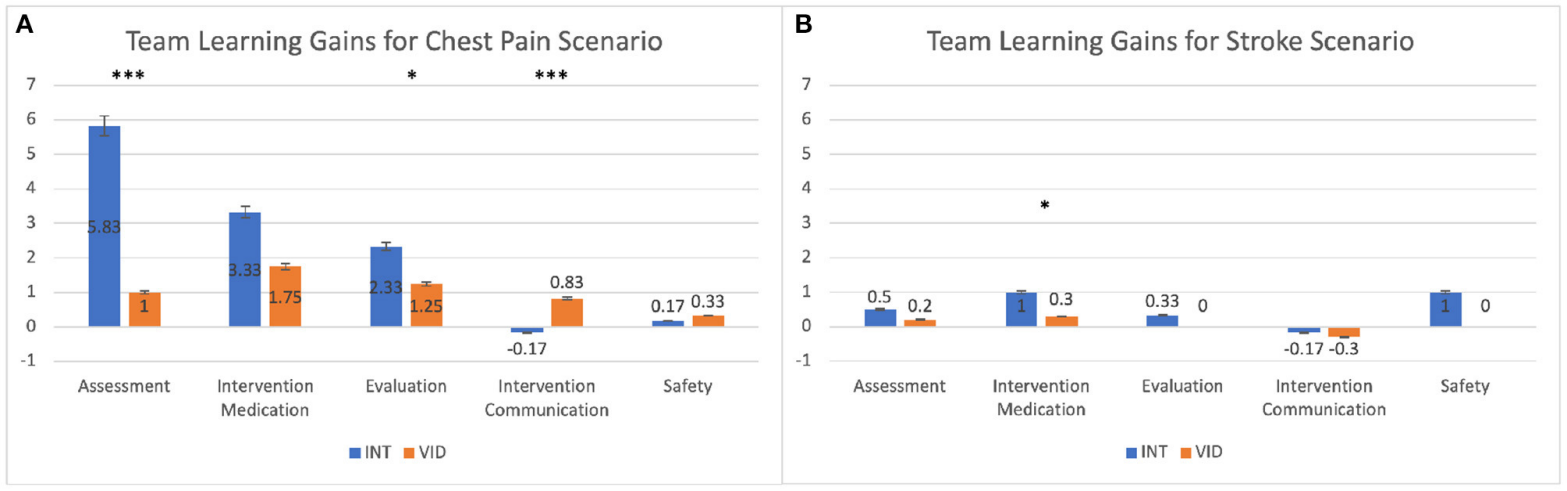
This graph compares the mean gains in participants' scores between the pre-questionnaire and the team response (team minus pre) for the INT and VID conditions. The results are shown for **(A)** the chest pain scenario and **(B)** the stroke scenario. *p ≤ 0.05; ***p ≤ 0.001. See details in Table 3.

In the chest pain scenario, we found that interactivity overall had a positive effect on students' learning. We calculated students' team learning gains by subtracting their pre-questionnaire scores from their team response scores and compared the mean gains for the INT and VID conditions. The team learning gains were greater for the INT condition than the VID condition in the A (*p* < 0.001) and E (*p* = 0.039) categories, and in the IM category, a trend (*p* = 0.059) suggested the same results. In the IC category, the team learning gains were greater for the VID condition than the INT condition (*p* = 0.001): the mean for the VID condition was positive (*M* = 0.83) while the mean for the INT condition was negative (*M* = −0.17). The results for team learning gains in the chest pain scenario are shown in Figure 2A and Table 3.

**Table 3:** This table shows the data for the mean gains in participants' scores between the pre-questionnaire and team response (team questionnaire).

**Team learning gains for chest pain scenario**						
**Category**	**W**	***p***	**Effect size**	**Condition**	**Mean**	**SD**
Assessment	0.5	<0.001	−0.99	INT	5.83	1.17
				VID	1.00	1.95
Intervention medication	16	0.059	−0.56	INT	3.33	0.52
				VID	1.75	1.82
Evaluation	15	0.039	−0.58	INT	2.33	0.52
				VID	1.25	1.14
Intervention communication	67	0.001	0.86	INT	−0.17	0.41
				VID	0.83	0.39
Safety	38	0.87	0.056	INT	0.17	0.41
				VID	0.33	0.99
**Team learning gains for stroke scenario**						
**Category**	**W**	***p***	**Effect size**	**Condition**	**Mean**	**SD**
Assessment	24.5	0.58	−0.18	INT	0.50	0.84
				VID	0.20	2.20
Intervention medication	12.5	0.039	−0.58	INT	1.00	0.63
				VID	0.30	0.48
Evaluation	–	–	–	INT	0.33	0.52
				VID	0.00	0.00
Intervention communication	28.00	0.87	-0.067	INT	−0.17	1.17
				VID	-0.30	1.16
Safety	20.5	0.32	−0.32	INT	1.00	1.27
				VID	0.00	2.06

*The statistical test for Evaluation in the stroke scenario could not be conducted because the variance of the means was equal to zero. This data is represented as graphs in Figure 2*.

**Figure 3:**
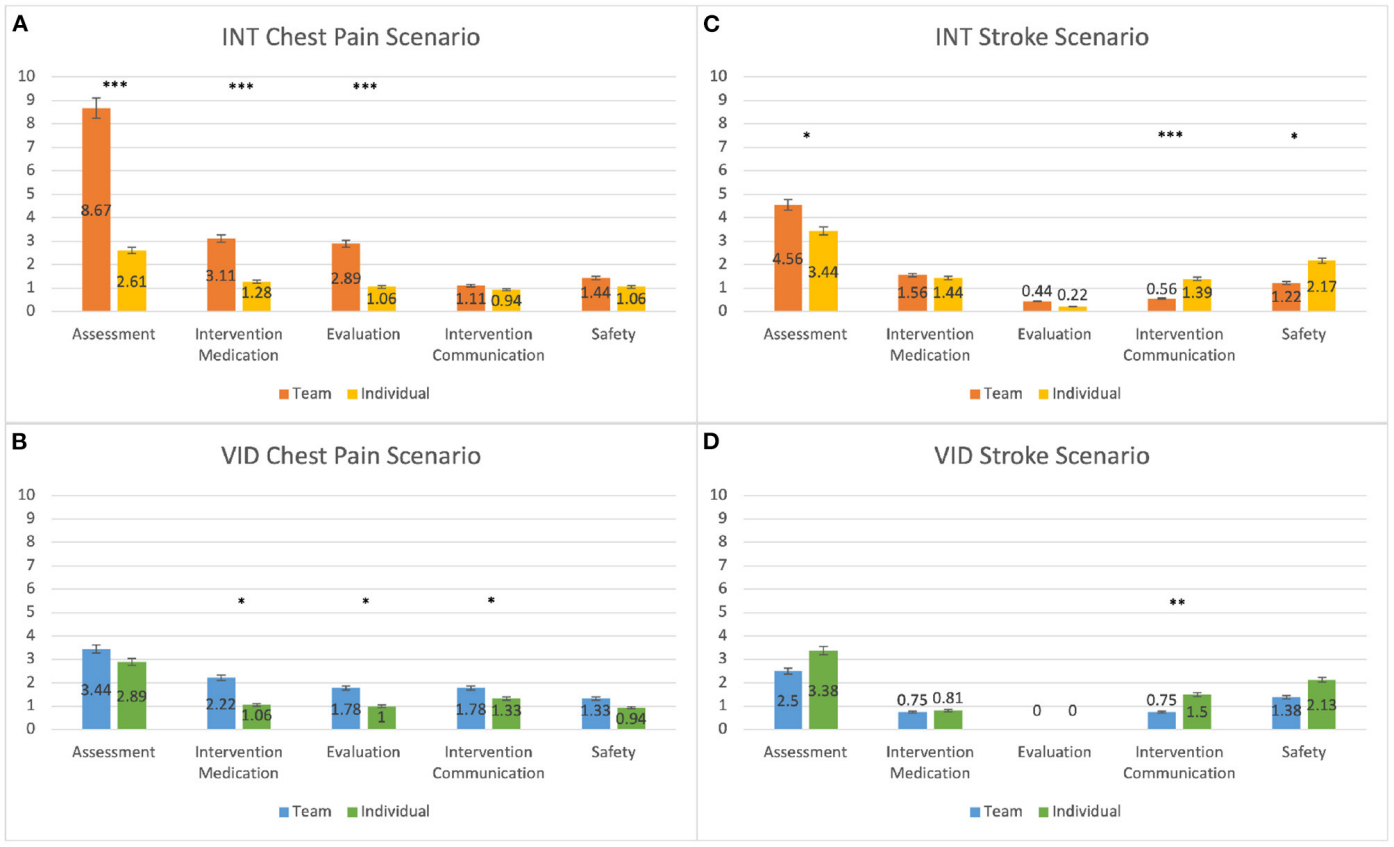
This graph compares participants' mean scores for the individual response (post-questionnaire) and the team response (team questionnaire). The results are shown for the **(A)** INT condition in the chest pain scenario, **(B)** VID condition in the chest pain scenario, **(C)** INT condition in the stroke scenario, and **(D)** VID condition in the stroke scenario. *p ≤ 0.05; **p ≤ 0.01; ***p ≤ 0.001. See details in Tables 4, 5.

We compared students' scores for the team response and individual response to determine the effect of teamwork on learning, and we found that teamwork had a positive impact on students' learning. For the INT condition, the team response scores were higher than the individual response scores in the A (*p* < 0.001), IM (*p* < 0.001), and E (*p* < 0.001) categories, and in the S category, a trend suggested the same results (*p* = 0.059). The results for the INT condition's team response and individual response scores in the chest pain scenario are shown in Figure 3A and Table 4. For the VID condition, the team response scores were higher than the individual response scores for the IM (*p* = 0.013), E (*p* = 0.011), and IC (*p* = 0.024) categories, and a trend in the S category suggested the same results (*p* = 0.097). The results for the VID condition's team response and individual response scores in the chest pain scenario are shown in Figure 3B and Table 4.

**Table 4:** This table shows the data for participants' mean scores for the individual response (post-questionnaire) and team response (team questionnaire) for the INT and VID conditions in the chest pain scenario.

**INT chest pain scenario**						
**Category**	**W**	***p***	**Effect size**	**Answer type**	**Mean**	**SD**
Assessment	0	<0.001	−1.00	Team	8.67	0.97
				Individual	2.61	1.20
Intervention medication	0	<0.001	−1.00	Team	3.11	0.32
				Individual	1.28	1.18
Evaluation	0	<0.001	−1.00	Team	2.89	0.32
				Individual	1.06	0.80
Intervention communication	8	0.30	−0.43	Team	1.11	0.32
				Individual	0.94	0.54
Safety	10	0.059	−0.64	Team	1.44	0.51
				Individual	1.06	0.87
**VID chest pain scenario**						
**Category**	**W**	***p***	**Effect size**	**Answer type**	**Mean**	**SD**
Assessment	13.5	0.17	−0.51	Team	3.44	1.76
				Individual	2.89	1.97
Intervention medication	13.5	0.013	−0.74	Team	2.22	1.59
				Individual	1.06	1.39
Evaluation	10	0.011	−0.78	Team	1.78	0.94
				Individual	1.00	0.97
Intervention communication	13	0.024	−0.67	Team	1.78	0.43
				Individual	1.33	0.69
Safety	6	0.097	−0.67	Team	1.33	0.69
				Individual	0.94	0.87

*This data is represented as graphs in Figures 3A,B*.

#### 3.1.2. Scenario 2: Stroke

For the stroke scenario, we found that interactivity promoted students' learning in the IM category. We compared students' team learning gains (team minus pre) and found that in the IM category, the gains were greater for the INT condition than the VID condition (*p* = 0.039). The results for the team learning gains in the stroke scenario are shown in Figure 2B and Table 3.

We found that the effect of teamwork on students' learning for the stroke scenario was mixed. For the INT condition, the team response scores were higher than the individual response scores in the A category (*p* = 0.027), however, in the IC and S categories, the individual response scores were higher than the team response scores (IC: *p* = 0.007; S: *p* = 0.015). The results for the INT condition's team response and individual response scores in the stroke scenario are shown in Figure 3C and Table 5. For the VID condition, the individual response scores were higher than the team response scores in the IC category (*p* = 0.007). The results for the VID condition's team response and individual response scores are shown in Figure 3D and Table 5.

**Table 5:** This table shows the data for participants' mean scores for the individual response (post-questionnaire) and team response (team questionnaire) for the INT and VID conditions in the chest pain scenario.

**INT stroke scenario**						
**Category**	**W**	***p***	**Effect size**	**Answer type**	**Mean**	**SD**
Assessment	17	0.027	−0.68	Team	4.56	1.82
				Individual	3.44	1.79
Intervention medication	22.5	0.63	−0.18	Team	1.56	0.71
				Individual	1.44	0.78
Evaluation	9	0.18	−0.50	Team	0.44	0.51
				Individual	0.22	0.43
Intervention communication	78	0.001	1.00	Team	0.56	0.71
				Individual	1.39	0.61
Safety	91	0.015	0.73	Team	1.22	1.44
				Individual	2.17	0.79
**VID stroke scenario**						
**Category**	**W**	***p***	**Effect size**	**Answer type**	**Mean**	**SD**
Assessment	58	0.14	0.49	Team	2.50	2.31
				Individual	3.38	2.06
Intervention medication	12	0.82	0.14	Team	0.75	0.86
				Individual	0.81	0.91
Evaluation	–	–	–	Team	0	–
				Individual	0	–
Intervention communication	45	0.007	1.00	Team	0.75	0.86
				Individual	1.50	0.63
Safety	36	0.12	0.60	Team	1.38	1.78
				Individual	2.13	1.26

*For the VID condition in the stroke scenario, the statistical test for the evaluation category could not be conducted because the variance of the means was equal to zero. This data is represented as graphs in Figures 3C,D*.

### 3.2. Survey

The following results describe participants' perceptions of teamwork and authenticity of the simulation. This data was collected through the SATTS and VPE questionnaires, respectively.

#### 3.2.1. Self-Assessment Teamwork Tool for Students (SATTS)

**Figure 4:**
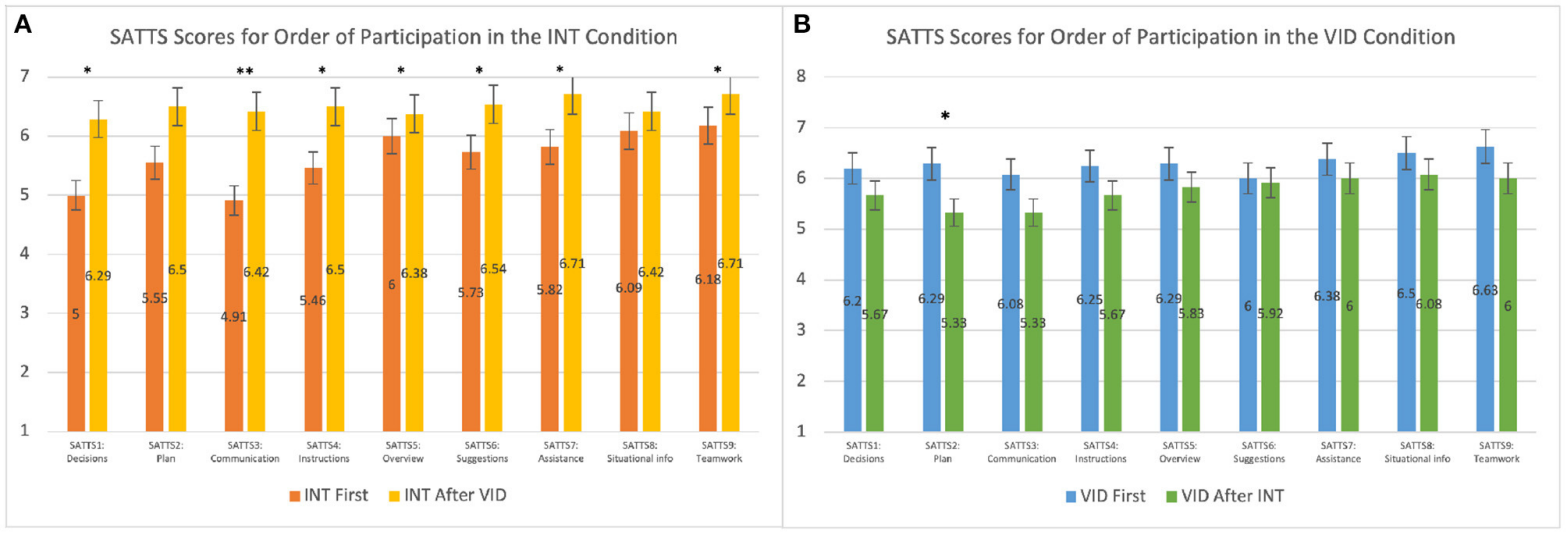
This graph compares the mean SATTS scores for order of participation in the **(A)** INT condition and **(B)** VID condition. *p ≤ 0.05; **p ≤ 0.01. See details in Table 6.

Results indicated that participants were more engaged in teamwork in the INT condition than the VID condition. We found that students who participated in the INT condition after the VID condition scored the INT condition higher on the SATTS questionnaire than students who participated in the INT condition first; the difference was statistically significant for all questions on the SATTS questionnaire except SATTS2 (Plan), which suggested a trend (*p* = 0.091), and SATTS8 (Situational info), which was not statistically significant (*p* = 0.11). The results for SATTS scores for the order of participation in the INT condition are shown in Figure 4A and Table 6. Students who participated in the VID condition after the INT condition scored the VID condition lower in SATTS2 (Plan) than students who participated in the VID condition first (*p* = 0.029). For SATTS9 (Teamwork), a trend suggested the same results (*p* = 0.08). The results for SATTS scores for the order of participation in the VID condition are shown in Figure 4B and Table 6.

**Table 6:** This table compares the mean SATTS scores for order of participation in the INT and VID conditions.

**SATTS scores for the order of participation in the INT condition**						
**Category**	**W**	***p***	**Effect size**	**Condition**	**Mean**	**SD**
SATTS1: Role	76	0.032	−0.42	INT first	5.00	2.10
				INT after VID	6.29	1.20
SATTS2: Plan	90.5	0.091	−0.31	INT first	5.55	1.97
				INT after VID	6.50	1.10
SATTS3: Communication	55	0.003	−0.58	INT first	4.91	1.92
				INT after VID	6.42	1.18
SATTS4: Instructions	68.5	0.012	−0.48	INT first	5.46	1.75
				INT after VID	6.50	1.10
SATTS5: Overview	80.5	0.04	−0.39	INT first	6.00	0.78
				INT after VID	6.38	1.31
SATTS6: Suggestions	79.5	0.032	−0.40	INT first	5.73	1.42
				INT after VID	6.54	1.02
SATTS7: Assistance	79.5	0.02	−0.40	INT first	5.82	1.78
				INT after VID	6.71	0.86
SATTS8: Situational info	92	0.11	−0.30	INT first	6.09	0.94
				INT after VID	6.42	1.14
SATTS9: Teamwork	89	0.04	−0.33	INT first	6.18	1.25
				INT after VID	6.71	1.04
**SATTS scores for the order of participation in the VID condition**						
**Category**	**W**	***p***	**Effect size**	**Condition**	**Mean**	**SD**
SATTS1: Role	103	0.15	−0.29	VID first	6.21	1.10
				VID After INT	5.67	1.16
SATTS2: Plan	82.5	0.029	−0.43	VID first	6.29	1.08
				VID After INT	5.33	1.72
SATTS3: Communication	103.5	0.16	−0.28	VID first	6.08	1.10
				VID after INT	5.33	1.61
SATTS4: Instructions	101.5	0.13	−0.30	VID first	6.25	1.07
				VID after INT	5.67	1.37
SATTS5: Overview	113.5	0.28	−0.21	VID first	6.29	0.96
				VID after INT	5.83	1.40
SATTS6: Suggestions	139	0.87	−0.035	VID first	6.00	1.14
				VID after INT	5.92	1.24
SATTS7: Assistance	123.5	0.46	−0.14	VID first	6.38	0.88
				VID after INT	6.00	1.41
SATTS8: Situational info	101.5	0.12	−0.30	VID first	6.50	0.89
				VID after INT	6.08	0.90
SATTS9: Teamwork	98.5	0.08	−0.32	VID first	6.63	0.71
				VID after INT	6.00	1.41

*The data is represented as graphs in Figure 4*.

#### 3.2.2. Virtual Patient Evaluation (VPE)

**Figure 5:**
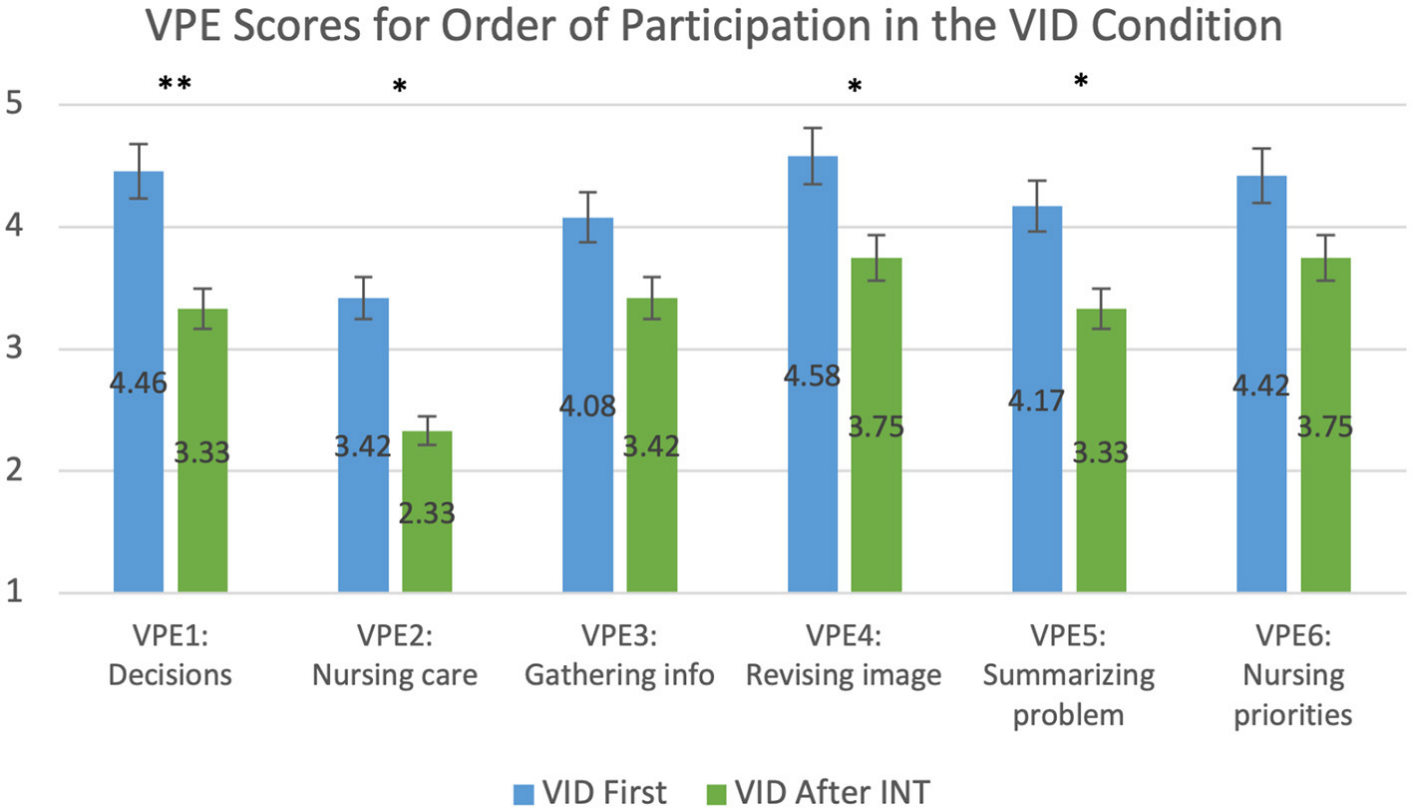
This graph compares the mean VPE scores of participants who were exposed to the VID condition first and those were exposed after the INT condition. *p ≤ 0.05; **p ≤ 0.01. See details in Table 7.

**Figure 6:**
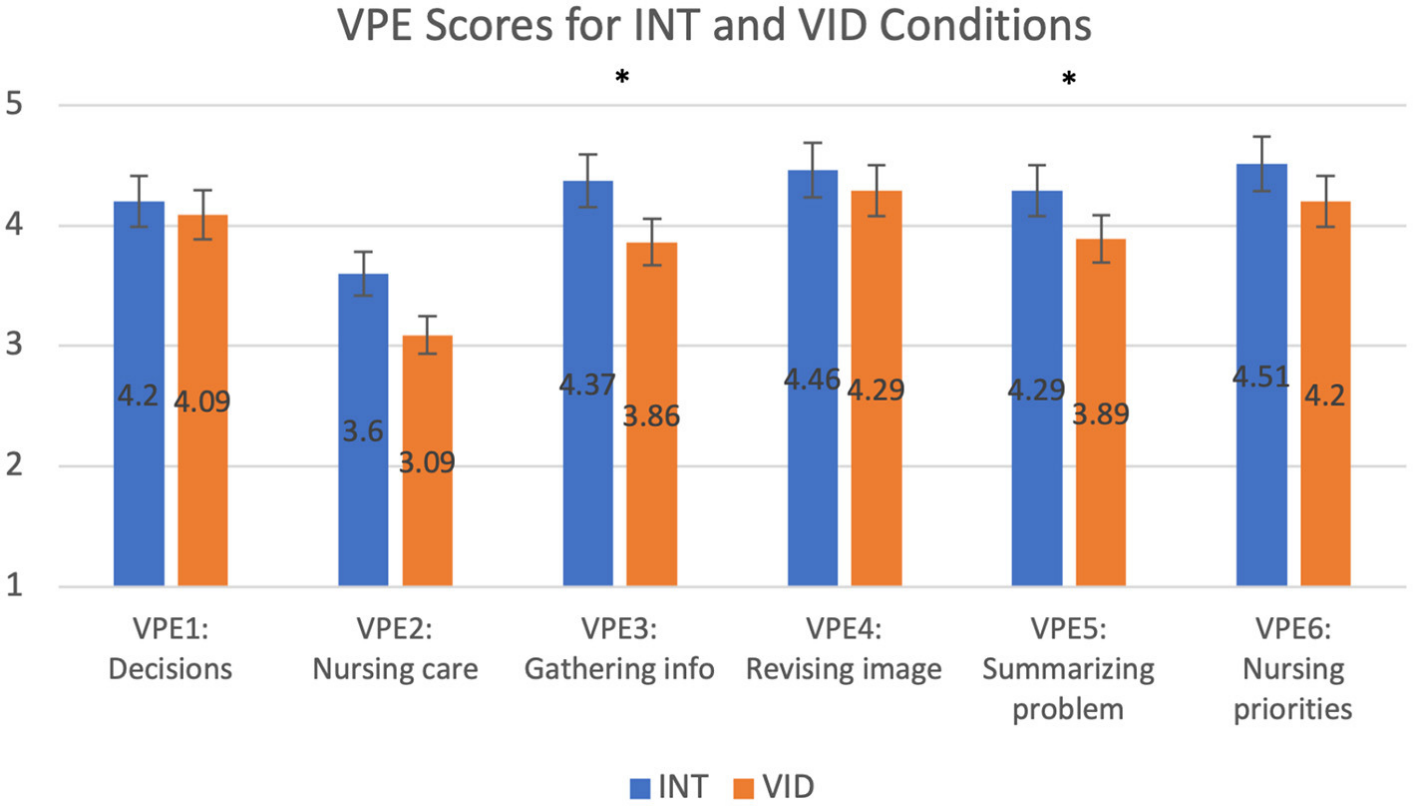
This graph compares the mean VPE scores for the INT and VID conditions. *p ≤ 0.05. See details in Table 8.

Participants perceived the INT condition to be more authentic than the VID condition. Students who participated in the VID condition after the INT condition scored the VID condition lower than students who participated in the VID condition first for all questions in the VPE questionnaire except for VPE3 (Gathering info) and VPE6 (Nursing priorities), which suggested trends (VPE3: *p* = 0.081; VPE6: *p* = 0.053). The results for VPE scores for the order of participation in the VID condition as shown in Figure 5 and Table 7. We did not find statistically significant results in the VPE scores for order of participation in the INT condition. In another statistical test, we found that students scored the INT condition higher than the VID condition on VPE3 (Gathering info) and VPE5 (Summarizing problem), regardless of the order of their participation; the difference was statistically significant for all questions on the VPE questionnaire except VPE6 (Nursing priorities), which suggested a trend (*p* = 0.084), and VPE2 (Nursing care), which was not statistically significant (*p* = 0.11). The results for VPE scores without accounting for the order of participation are shown in Figure 6 and Table 8. Lastly, we found that students' average VPE scores were higher for the INT condition (*M* = 4.24, SD = 0.918) than the VID condition (*M* = 3.90, SD = 0.959). These results were statistically significant (W = 299.500, *p* = 0.029).

**Table 7:** This table compares the mean VPE scores of participants who were exposed to the VID condition first and those were exposed after the INT condition.

**Category**	**W**	***p***	**Effect size**	**Condition**	**Mean**	**SD**
VPE1: Decisions	64.5	0.005	−0.55	VID first	4.46	0.72
				VID after INT	3.33	1.23
VPE2: Nursing care	86.5	0.049	−0.40	VID first	3.42	1.50
				VID after INT	2.33	1.23
VPE3: Gathering info	94	0.081	−0.35	VID first	4.08	1.21
				VID after INT	3.42	1.24
VPE4: Revising image	83	0.022	−0.42	VID first	4.58	0.72
				VID after INT	3.75	1.22
VPE5: Summarizing problem	82	0.031	−0.43	VID first	4.17	0.96
				VID after INT	3.33	1.07
VPE6: Nursing priorities	90	0.053	−0.38	VID first	4.42	0.78
				VID after INT	3.75	1.06

*The data is represented as a graph in Figure 5*.

**Table 8:** This table compares the mean VPE scores for the INT and VID conditions, regardless of order of participation.

**Category**	**W**	***p***	**Effect size**	**Condition**	**Mean**	**SD**
VPE1: Decisions	115	0.42	0.21	INT	4.20	1.16
				VID	4.09	1.07
VPE2: Nursing care	122.5	0.11	0.43	INT	3.60	1.48
				VID	3.09	1.50
VPE3: Gathering info	135.5	0.025	0.59	INT	4.37	0.91
				VID	3.86	1.26
VPE4: Revising image	76.5	0.35	0.28	INT	4.46	1.07
				VID	4.29	0.99
VPE5: Summarizing problem	143.5	0.046	0.51	INT	4.29	1.05
				VID	3.89	1.08
VPE6: Nursing priorities	90	0.084	0.50	INT	4.51	0.82
				VID	4.20	0.93

*The data is represented as a graph in Figure 6*.

### 3.3. Qualitative Feedback

Out of the 36 participants, 23 participants preferred the INT condition, 11 participants preferred the VID condition, and 2 participants did not specify a preference. Seven participants preferred the INT condition because it was more engaging than the VID condition. Three participants mentioned that they preferred the INT condition because they had input in the sequence of steps. Three participants mentioned that it was helpful to receive feedback about their patient care decisions. Two participants preferred the INT condition because it required more critical thinking than the VID condition.

Out of the 11 participants that preferred the VID condition, three mentioned that it was unclear how to use the INT simulation technology. Two participants mentioned that the videos played in the INT condition were lagging. Two participants mentioned that they preferred to watch the nurse perform the full scenario in the correct sequence of steps.

## 4. Discussion

The QSEN approach to categorizing the data allowed the researchers to identify deep learning through systems thinking and critical thinking (26). Critical thinking is identified using the QSEN categories and completing the nursing process (27). Using consensus, we ranked the QSEN competencies based on the nature of the scenarios. The A, IM, and E competencies were ascribed greater weight since they required decision-making opportunities to interpret data, select correct medication and evaluate patient changes. We determined that the A, IM, and E categories constitute higher order learning and the IC and S categories constitute lower order learning. Additionally, the chest pain and stroke scenarios had differences in their levels of complexity, which is important to note when evaluating the results. The chest pain scenario required students to make more decisions and challenged them to determine the cause of the patient's pain and intervene appropriately, while the focus of the stroke scenario was the neurological assessment, which required fewer decisions and interventions. We evaluated whether the complexity of the scenario influenced the effects of interactivity.

**Learning and Interactivity**

We observed that higher order learning is promoted more through interactive simulation than non-interactive simulation. In our study, statistically significant results in the higher order learning categories showed that between the pre-questionnaire and team response, students had greater learning gains in the INT condition than the VID condition. IC was the only category for which students' scores were higher in the VID than the INT condition; in fact, the gains for the INT condition in this category were negative. We designed the interactivity to be focused on decisions as opposed to the manikin's responses for both the INT group and the VID group. By design of the intervention, communication was not supported during the care of the patient which might have created a situation where the students did not expect to get a response from the manikin and therefore did not engage in purposeful communication. We believe that this resulted in the decline of students' scores in the IC category. It is also interesting to note that the participants in the VID condition had watched the full solution to the scenario prior to responding to the questionnaires while students in the INT condition had no prior exposure to the solution. Despite this, participants in the INT condition overall demonstrated more significant higher order learning than those in the VID condition. This further demonstrates that interactivity promotes higher order learning more than non-interactivity. These results support our first research question (Q1).

**Learning and Teamwork**

Teamwork was shown to have a positive impact on students' higher order learning, but on lower order learning teamwork had a lesser impact. In our results, there were statistically significant differences for all three higher order learning categories between students' individual and team response scores, with the team response scores being higher. However, in the stroke scenario, we found that for the lower order learning categories (IC and S), individual work produced higher scores than teamwork. These results answer our second research question (Q2).

**Perception of Authenticity**

Interaction with the simulation system was shown to greatly increase perceptions of authenticity. Participants felt more actively engaged in gathering information (VPE3) and summarizing the problem (VPE5) in the INT condition than the VID condition. A trend also suggested that participants may have felt more actively engaged in thinking about nursing priorities (VPE6) in the INT condition than the VID condition. Additionally, participants' mean scores in the VPE questionnaire were overall higher for the INT condition than the VID condition. With the added interactive component, the simulation experience was perceived as largely more authentic than without interactivity. These results support our third research question (Q3).

**Order of Participation**

We found that the order of participation in the INT and VID conditions affected students' perceptions of the simulation. Students who experienced the VID simulation before the INT simulation perceived teamwork and authenticity in the INT simulation as greater than students who were exposed to the INT simulation first. Similarly, when students were exposed to the INT simulation before the VID simulation, they perceived teamwork and authenticity in the VID simulation to be lower than students who were exposed to the VID simulation first. Participants compared their initial simulation experience to their subsequent experience, which may suggest that the VID simulation comparatively generated a lesser sense of teamwork and perception of authenticity than the INT simulation. With these results, we found that the order of participation does affect perceptions of the simulation: students perceive both teamwork and authenticity to be highest when they are exposed to the VID simulation first and then the INT simulation. This supports our fourth research question (Q4).

Based on these results, we recommend that non-interactive video simulation is supplemented with interactive simulation. To promote higher order learning, we recommend that complex scenarios are used with teamwork activity. To promote lower order learning, we recommend that simple scenarios and are used with individual activity. The non-interactive simulation should be conducted before the interactive simulation to increase perceptions of teamwork and authenticity. This will enable comprehensive learning of both higher order and lower order knowledge and increase students' perceptions of the simulation experience.

The IVS software has demonstrated to be a valid remote simulation technology. The technology embraces three pedagogically sound strategies in simulation education: (1) The participant observer role, (2) teamwork, and (3) productive failure (PF). There is work to support the use of participant observers in simulation and evidence to suggest that learning outcomes such as clinical judgement, insight, and conceptual thinking can be achieved by viewing a simulation (28). Likewise, team-based learning has been used with much success in courses such as health assessment and with simulation (29). Team-based learning has been found to develop nursing students' teamwork and collaborative skills as they interact with one another (30). The IVS technology harnesses the power of remote availability and teamwork to enhance critical thinking skills. At various points in the simulation, the video is paused and students choose their next steps without being given options; in essence allowing the student to think critically before choosing the next action without showing them the answer. Showing the answer during the simulation can inadvertently make the activity easier or harder than necessary before giving the students the opportunity to think. Palominos et al. (31) established the concept of PF, an approach to simulation learning that emphasizes the educational value of making mistakes in a non-threatening environment. The premise of PF is to allow learners to make errors and then follow their error experiences with opportunities to identify the correct solution. By completing the pre-questionnaire prior to the simulations, students gained exposure to the SBAR and were able to plan their patient care interventions ahead of time. This allowed the students to attempt the solution despite the possibility of errors and prepared them to reconsidered their interventions at later points in the simulations, thus facilitating their learning. The INT condition implemented a reinforced PF strategy: in the simulation, students needed to rethink their decisions before communicating their steps to the facilitator. The INT condition promoted greater learning in the higher order categories and this may be attributed to the reinforced PF strategy. Additionally, in both conditions, the students had the opportunity to collaborate in pairs before finalizing their answers to the scenarios. The design of the study allowed students to make mistakes and then guided them to identify their errors before being debriefed by the facilitator.

During the pandemic, healthcare students' exposure to training has been maintained largely through video-based resources (32). The IVS software engages students in interactive simulation experiences remotely and while using educators' existing video content. By making simulation content more interactive, we are extending existing resources, allowing for the development of critical thinking and decision-making. The IVS technology allows for greater accessibility for students and faculty in remote environment.

### 4.1. Limitations

A notable feature of the software is that it allows instructors to upload their own content and customize the simulation to their preferences. For this study, the software development was focused on the functionality and display of the simulation, and as a result, the graphical user interface (GUI) for uploading content was not fully developed. This did not affect the execution of the study as the dashboard of control buttons and the students' video display were fully developed. Also, since the INT simulation was conducted over Zoom, the frame rate of the videos was reduced, causing lagging at times. Some participants noted this in their surveys, saying that it negatively affected their simulation experience. The INT simulation was also controlled by the facilitator, making students' interaction with the software indirect. Additionally, the study was designed to evaluate students' implementation of patient care and knowledge of protocols and did not evaluate communication skills. The simulation videos showed the nurse communicating with a manikin rather than an actual patient, with the patient's dialogue being displayed as captions in the videos, and in the INT condition, the interaction occurred between the student and simulation system and did not involve the patient. As a result, the study design was not optimal for students' learning of IC competencies. Lastly, in a typical simulation, the debrief would be longer than 10 min but due to limitations of scheduling and timing the debrief was shortened. Ideally, more time should be allocated for the debrief but the reduced debrief time did not affect the results of the study.

### 4.2. Future Work

We are planning to further develop the software's GUI to improve the usability and customizability of the software for educators. Additionally, streaming the videos over Zoom caused lagging and choppiness that negatively affected some students' experiences during the simulation. We intend to develop the software so that it is not dependent on Zoom; rather, the videos could be stored locally on students' computers instead of being streamed over a video conferencing application. This would eliminate the problem of lagging, providing a better simulation experience to students. We also intend to develop features that will eliminate the need for a facilitator to mediate the simulation for students, allowing the students to interact with the software directly and independently if desired.

#### 4.2.1. Automated After-Action Review

Debrief is essential to the development of students' skills in healthcare simulation. In a debrief, the facilitator guides learners to reflect on their performance in a discussion that examines various aspects of the simulation exercise. After-action review (AAR) is a structured methodology for debriefing that was initially developed by the U.S. Army but has been adopted in healthcare simulation (33). As we further develop the IVS software, we intend to create an automated AAR in which the system would provide feedback at the end of the simulation to indicate where students' actions were correct or incorrect. The automated AAR can be used to guide the facilitator's debrief and provide students the option to practice simulation exercises independently with standardized feedback.

## 5. Conclusion

Through the COVID-19 pandemic, it became apparent that traditional manikin simulation has limitations, namely those of safety and flexibility. As a replacement for manikin simulation, many nursing educators resorted to videos to teach their learners remotely; however, without interactivity, the simulation does not meet the interactivity standards of best practice for healthcare simulation. By comparing the INT condition to the VID condition, we evaluated the effect of interactivity on students' learning and perceptions of the simulation. We found that interactive simulation promotes students' learning and is perceived as largely more authentic than non-interactive simulation. We also found that higher level learning is promoted more through teamwork while lower level learning is promoted more through individual work. Lastly, we found that students' order of participation in interactive and non-interactive simulation affects their perceptions of the experience; perceptions of teamwork and authenticity are increased when students participate in non-interactive simulation before interactive simulation, though this also indicates that students perceive teamwork and authenticity to be comparatively better in the interactive simulation than the non-interactive simulation. Given these findings, we recommend that non-interactive simulation is supplemented by interactive simulation to promote comprehensive learning and increase students' perceptions of the experience. We recommend that educators use teamwork for complex scenarios to promote higher level learning, and individual work for simple scenarios to promote lower level learning. Future research into remote simulation options should strive to limit dependence on facilitators and other platforms, allowing students to practice scenarios independently and with a standardized after action review.

## Data Availability Statement

The raw data supporting the conclusions of this article will be made available by the authors, without undue reservation.

## Ethics Statement

The studies involving human participants were reviewed and approved by IRB at University of Central Florida. The participants provided their written informed consent to participate in this study.

## Author Contributions

The study was designed by all authors. LG and HP provided nursing education expertise and collected the data. SD and DM developed the IVS software and analyzed the data. The manuscript was written by DM, LG, HP, and SD. SD supervised the project and provided extensive comments and revisions to the manuscript. All authors contributed to the article considerably and deserve authorship, reviewed the article, and approved the submitted version.

## Conflict of Interest

The authors declare that the research was conducted in the absence of any commercial or financial relationships that could be construed as a potential conflict of interest.

## Publisher's Note

All claims expressed in this article are solely those of the authors and do not necessarily represent those of their affiliated organizations, or those of the publisher, the editors and the reviewers. Any product that may be evaluated in this article, or claim that may be made by its manufacturer, is not guaranteed or endorsed by the publisher.
